# Development of Calcium Signaling in Postsynaptic Neurons of the Medial Nucleus of the Trapezoid Body in the Mouse

**DOI:** 10.1007/s10162-026-01066-8

**Published:** 2026-08-03

**Authors:** Tasmuna T. Tanmy, Huimei Wang, Yong Lu

**Affiliations:** 1 Department of Biomedical Sciences, College of Medicine, University Hospitals NEOMED Hearing Research Center, Northeast Ohio Medical University, Rootstown, OH 44272 USA; 2 School of Biomedical Sciences, Kent State University, Kent, OH 44242 USA

**Keywords:** Medial nucleus of trapezoid body, Calcium imaging, Neuronal development, Glutamate receptors, Sound localization circuit

## Abstract

**Purpose:**

In the central nervous system, Ca^2^⁺ signaling plays a pivotal role in various cellular processes, including neuronal development and maturation. Disruption of Ca^2^⁺ homeostasis impairs auditory circuit formation, compromising auditory processing and behavior. Here, we aimed to investigate systematically the development of Ca^2^⁺ signaling in neurons of the medial nucleus of the trapezoid body (MNTB), a brainstem nucleus critical for sound localization.

**Methods:**

Using Ca^2^⁺ imaging in brain slices from mice genetically expressing the Ca^2^⁺ indicator GCaMP in glycinergic cells, we studied Ca^2^⁺ signaling in MNTB neurons from mice of either sex at three developmental milestone stages, in response to both bath-applied agonists of various neurotransmitter receptors and synaptic activation of glutamatergic afferents.

**Results:**

Prior to hearing onset at postnatal 7 days (P7), robust Ca^2^⁺ responses were evoked in MNTB neurons upon activation of glutamate receptors (NMDARs, AMPARs, and group I (Gp-I) mGluRs by 200 μM NMDA, 100 μM AMPA, and 200 μM 3,5-DHPG, respectively), whereas after hearing onset (P14 and P21), responses induced by NMDA and 3,5-DHPG declined markedly while AMPA-induced responses remained relatively strong. Ca^2^⁺ responses upon application of GABA (100 μM) and glycine (200 μM) were detected in neonatal mice, diminished in a few days after birth, and almost completely disappeared by P7. Whole-cell patch-clamp recordings showed that stimulation of excitatory afferents evoked action potentials across all ages with no differences in firing frequency up to 100 Hz, but Ca^2^⁺ responses varied in a stimulus intensity- and frequency-dependent manner and exhibited developmental downregulation.

**Conclusion:**

Ca^2^⁺ signaling induced by activation of the major transmitter receptors in MNTB neurons is highly developmentally down-regulated.

## Introduction

In the nervous system, Ca^2^⁺ generate versatile intracellular signals, serving as key regulators of numerous cellular processes [1], including neuronal development and maturation [2]. Ca^2^⁺ regulates neuronal gene expression, membrane excitability, synaptogenesis, synaptic transmission, energy metabolism, and higher functions such as learning and memory [3–5]. It also mediates synaptic plasticity, including both long-term potentiation and long-term depression [6]. Intracellular free Ca^2+^ concentration is tightly controlled by a multitude of mechanisms [7]. Disruption of Ca^2^⁺ homeostasis and signaling during neurodevelopment results in profound physiological deficits, including impaired cognitive function and increased susceptibility to neurological disorders such as autism spectrum disorder [8], Alzheimer’s disease [9], epilepsy [10] and schizophrenia [11]. For the auditory system, disruption of Ca^2+^ homeostasis results in abnormal auditory circuit development, leading to compromised auditory processing and behavior [12].

The medial nucleus of the trapezoid body (MNTB) is a key component of the superior olivary complex, essential for sound localization [13,14]. Principal MNTB neurons receive strong excitatory input from globular bushy cells in the contralateral cochlear nucleus via the calyx of Held, as well as additional excitatory and inhibitory inputs primarily from other brainstem nuclei [15]. In turn, MNTB provides powerful glycinergic inhibition to multiple auditory centers, including the lateral and medial superior olive and other brainstem nuclei [16,17]. Through this inhibitory output, the MNTB shapes auditory circuit activity, enabling precise sound localization and temporal processing [18]. As a critical hub in the auditory brainstem pathway for sound localization, the MNTB undergoes key developmental processes including neuronal survival and death, synapse maturation, neurotransmission, and signal transduction, all of which depend on Ca^2^⁺ signaling mediated by a variety of pathways. Studying Ca^2^⁺ signaling in the MNTB during early development is essential for understanding how precise auditory circuits are established, maintained, and functional [19].

Ca^2+^ signaling in neurons receiving glutamatergic inputs is mediated primarily by Ca^2+^-permeable receptor channels such as NMDA receptors (NMDARs) and AMPA receptors (AMPARs), voltage-gated Ca^2+^ channels (VGCCs), and release of Ca^2+^ from internal stores [20]. Age-dependent changes in NMDAR expression at the calyx of Held–MNTB synapse have been reported, with receptor levels downregulated after hearing onset and NMDAR-EPSC amplitude declining thereafter [21–23], suggesting a downregulation of their Ca^2+^ signaling [24]. The existing studies on receptor-mediated Ca^2^⁺ signaling, however, are scattered and typically associated with single-cell recordings [19,25–27]. There is a lack of systematic account on various receptor channels from birth to maturation at the cell population level. This gap limits our understanding of the mechanisms governing Ca^2+^ signaling during the development of the sound localization circuits. Because Ca^2^⁺ influx occurs via diverse plasma membrane receptors, we proposed a systematic approach to study the development of Ca^2+^ signaling associated with each of the major receptor types. The principal MNTB neurons are glycinergic and their large calyceal inputs are glutamatergic. By utilizing a glycine transporter 2 (GlyT2)-Cre-driven expression of a Ca^2+^ indicator GCaMP, we were able to monitor the Ca^2+^ signaling in specifically postsynaptic MNTB neurons without potential presynaptic Ca^2+^ signaling contamination when bath loading of Ca^2+^ indicator dyes was used [26,27]. We hypothesize that the overall Ca^2^⁺ signaling in MNTB neurons is developmentally downregulated with different developmental trajectories depending on the transmitter receptor.

## Materials and Methods

### Animals and Ethical Approval

Glycine Transporter 2-Cre (GlyT2-Cre, #030730-UCD) mice were originally purchased from the mutant mouse resource and research centers (MMRRC) supported by NIH and were crossed with the reporter line ROSA26-tdTomato/GCaMP6f mice, which were purchased from the Jackson Laboratory (#031968). Genotype was confirmed with standard PCR protocol provided by the Jackson Laboratory, and genotyping was performed by Transnetyx (Cordova, TN). Mice were housed in a vivarium with a normal light–dark cycle (12 h light and 12 h dark). The offspring inherits the driver-reporter combination of genes (GlyT2-Cre/tdT-GCaMP6f) and allows Cre-dependent expression of the genetically encoded Ca^2^⁺ indicator protein GCaMP6f, which is used to monitor intracellular Ca^2+^ dynamics in response to receptor activation in MNTB glycinergic neurons, as done in other tissue types [28–30]. The mouse line also allows Cre-dependent expression of tdTomato in glycinergic neurons such as the principal neurons in MNTB. All animal procedures were approved by the Institutional Animal Care and Use Committees at the Northeast Ohio Medical University (NEOMED, Rootstown, OH), and were performed in accordance with the National Institutes of Health policies and the ARRIVE guidelines on animal use.

### Brain Slice Preparation

Coronal brainstem slices (200—250 μm in thickness) were prepared from 0–3 weeks old mice of both sexes, as described in our previous publications [31,32]. Specifically, mice at ages of postnatal (P) 7 and 8 days were defined as the P7 group. The P14 group included mice at ages of P14 and P15, and the P21 group included P21 and P22 mice. Additionally, mice at ages of P1, P2, P3, and P7 were used in the experiment in which inhibitory receptor-mediated Ca^2^⁺ signaling was studied. Mice were deeply anesthetized with isoflurane and rapidly decapitated. The brainstem was removed and sliced in ice-cold low-Na^+^ artificial cerebrospinal fluid (ASCF) containing the following (in mM): 250 glycerol, 3 KCl, 1.2 KH_2_PO_4_, 20 NaHCO_3_, 3 HEPES, 0.12 CaCl_2_, 5 MgCl_2_, and 10 glucose, pH 7.4 (when gassed with 95% O_2_ and 5% CO_2_) and osmolarity of ~ 290 mmol/kg. Slices were incubated in an interface chamber at 34–36 °C for ~ 1 h in normal ACSF containing the following (in mM): 130 NaCl, 3 KCl, 1.2 KH_2_PO_4_, 20 NaHCO_3_, 3 HEPES, 1.2 CaCl_2_, 1.3 MgSO_4_, and 10 glucose, pH 7.4 and osmolarity of ~ 290 mmol/kg. The interface chamber was covered with aluminum foil to ensure that the slices were shielded from bright room illumination, because GCaMP indicators are light sensitive and bleach if exposed to intense light [33]. For recording, slices were transferred to a 0.5 mL chamber mounted on an Olympus BX51W1 fixed-stage upright microscope with a 40 × water-immersion objective (LUMPlan FLN 40x/0.80w ∞/0/FN26.5). The chamber was continuously superfused with ACSF (2–3 mL/min) driven by gravity. Recordings were performed under near physiological temperatures (34–36 °C) maintained with automatic heater controller TC-324B (Warner Instruments Corporation, Holliston, MA).

### Calcium Imaging

Coronal brainstem slices were collected from the GlyT2-Cre-tdT/GCaMP6f mice and placed in the microscope recording chamber and secured with a harp made of platinum and nylon strings. The MNTB was identified with a 10 × air objective and imaged with the water-immersion objective at 40 × magnification. Adapting the calcium imaging protocol from a previous study [34], the neurons were illuminated using a light-emitting diode light source (pE300^ultra^ LED; CoolLED, UK) filtered for blue light excitation (467–498 nm, beam splitter DM 506 nm, emission BA 515–556 nm) at the lowest intensity setting that provided reasonable signal strength. The presence of green GCaMP expression was confirmed in the MNTB neurons. Epifluorescence was collected using an Andor Zyla 4.1 sCMOS camera (Oxford Instruments, UK) with high sensitivity and low noise. LED and camera were controlled and synchronized with the Micromanager 2.0 software [35]. For mouse litters that were genotyped, the pups determined as Cre positive by Transnetyx (Cordova, TN) were used. For mouse litters that were not genotyped, the mice with detection of red fluorescence in MNTB neurons under the light excitation (530–550 nm, beam splitter DM 570 nm, emission BA 590 nm) were used because the tdT/GCaMP6f line expresses tdTomato in glycinergic cells besides the green GCaMP signal. The room lights were minimized, and the microscope cage was covered with a black cloth throughout the recording session to prevent the fluorescent signal from bleaching over time. The camera exposure time was set at 100 ms with binning (grouping of camera pixels before camera readout) 1 × 1. After a 15-min stabilization period in the recording chamber, a focal plane was chosen that includes a cluster of multiple fluorescent MNTB cell bodies, which were marked as the region of interest (ROI). Then the multi-dimension acquisitions were performed to record the Ca^2^⁺ signals. For the experiments of bath application of drugs, 200 images were acquired at the interval of 3 s, with a total of 10 min of recordings. For a small number of experiments when both the agonist and antagonist for a certain type of receptors were applied, the imaging period lasted for up to 30 min. For the electrical stimulation experiments, the frame rate was increased to 1 Hz (interval of 1 s), in order to record the faster Ca^2^⁺ events. The live profile of the Ca^2^⁺ dynamics was monitored using the real time intensity plots and the images were saved as stack files for offline analyses. A minority of the early recordings were performed using the Tillvision Imaging System v4.5 (Till Photonics, Germany), which consisted of a Polychrome 5000 with its control unit ICU as the illumination source and an IMAGO CCD camera (Sensor type: Scientific CCD, monochrome; common active resolution: ~ 1376 × 1040 pixels).

It is worth noting that the GlyT2-Cre mouse line obtained from the MMRRC exhibits unknown levels of Cre recombinase activity. Variability in Cre activity can influence the expression levels of tdT/GCaMP6f, resulting in differing fluorescence intensities of the tdTomato (red) signal in glycinergic cells and the GCaMP (green) signal. In addition, the overall fluorescence intensity of both tdTomato and GCaMP showed a tendency to decline with increasing age of the animals. To minimize variability in Ca^2^⁺ signaling arising from these factors, we consistently used the minimal excitation light intensity necessary to record Ca^2^⁺ signals across tissues from different ages, thereby achieving comparable baseline fluorescence levels.

### In Vitro Whole-cell Patching Recording

Patch pipettes were drawn on a PP-830 or PC-100 Microelectrode Puller (Narishige, Japan) to a 1–2 µm tip diameter using borosilicate glass micropipettes (inner diameter, 0.84 mm; outer diameter, 1.5 mm, World Precision Instruments, Sarasota, FL). The electrodes had resistances between 3 and 6 MΩ when filled with internal solution. The internal solution for action potential (AP) recordings in current clamp mode contains the following (in mM): 130 K-gluconate, 4.5 MgCl_2_, 4.4 Tris-phosphocreatine, 9 HEPES, 5 EGTA, 4 Na-ATP, 0.48 Na-GTP, with pH 7.3, adjusted with KOH, and osmolarity around 290 mmol/kg. The liquid junction potential for the AP recording solutions was—9.7 mV, and data were corrected accordingly. Current-clamp experiments were performed with an AxoClamp 2B amplifier (Molecular Devices, San Jose, CA). Recordings were performed under near physiological temperatures (34–36 °C) and obtained at resting membrane potential (RMP). Data were low-pass filtered at 3 or 5 kHz and digitized with a Data Acquisition Interface ITC-18 (InstruTech, Longmont, CO) at 50 kHz. Recording protocols were written and run using the acquisition and analysis software AxoGraph X (AxoGraph Scientific, Australia).

### Afferent Stimulation of the Trapezoid Body

Extracellular stimulation of afferent axons was performed using a concentric bipolar electrode with a tip core diameter of 127 µm (World Precision Instruments, Sarasota, FL). For the synaptic activity-induced Ca^2^⁺ responses in MNTB, the stimulating electrode was placed with a Micromanipulator NMN-25 (Narishige, Japan) and was positioned at the ventral brainstem midline to activate the excitatory afferent fibers innervating MNTB from the contralateral cochlear nucleus. A train stimulation (10, 50, and 100 Hz, duration of 5 s) was delivered through a Stimulator A320RC (World Precision Instruments, Sarasota, FL) controlled by a Train/Delay Generator DG2A (Digitimer Ltd., England). The stimulus intensity (0.5—0.7 mA) at which stable synaptic response was elicited was chosen for experiments. The RMP was determined immediately after whole-cell mode was established. APs were detected using a threshold method by setting the threshold at −20 mV for event detection. Not all MNTB neurons had an overshoot (above 0 mV) in their AP waveforms, but all of them had a peak amplitude more positive than −20 mV. The spike firing frequency across different age groups under various stimulation frequencies was compared.

### Drug Application

All drugs were delivered via bath application. The drugs were prepared at their respective working concentrations, which were at least threefold their individual EC_50_. This was expected to achieve a saturating concentration and thus full activation of the respective receptors in our experiments. α-amino-3-hydroxy-5-methyl-4-isoxazolepropionic acid (AMPA; 100 µM) (EC_50_ ~ 0.1—11 µM) and N-methyl-D-aspartate (NMDA; 200 µM) (EC_50_ ~ 1—50 µM) were used to activate AMPARs and NMDARs, respectively. Glycine (200 µM) was added to the NMDA stock solution to act as the co-factor for NMDARs. 6,7-Dinitroquinoxaline-2,3-dione (DNQX; 50 µM) and D-2-amino-5-phosphonopentanoic acid (AP5, 50 µM) were used to block AMPARs and NMDARs, respectively. To activate Gp-I mGluRs, 3,5-Dihydroxyphenylglycine (3,5- DHPG) was used at a working concentration of 200 µM (EC_50_ 0.7–60 µM) [36]. GABA (100 µM) and Glycine (200 µM) were used as a combination to activate GABA_A_Rs and GlyRs in neonatal mice. All chemicals were purchased from Sigma-Aldrich except for 3,5-DHPG, which was obtained from Tocris Bioscience (R&D Systems, Minneapolis, MN). From each mouse, depending on their age, 2–3 coronal brainstem slices were collected containing portions of the MNTB. Individual slices were used only once for each agonist. We have used only one slice per animal to report one data point for each drug (*N =* number of animals).

### Data Analyses

For calcium imaging, GCaMP6f fluorescence image time-series were processed using Micromanager and Image J software. ROIs were drawn around individual, in-focus fluorescent cells from a single focal plane. The baseline fluorescence varied substantially in amplitude across neurons, as reflected in the raw fluorescence intensity values (Table 1). To ensure statistical rigor, mean fluorescence intensity within each ROI was measured for each frame, using the added plugins program Time Series Analyzer V2.0. Data from 8–12 individual fluorescent cells at about the same focus plane within the ROI were collected to calculate the mean fluorescent intensity. They were then normalized by setting the initial response at 100%. For each ROI, normalized peak amplitude (%) is the maximal percent change in Ca^2^⁺ signaling in response to drug applications or electrical stimulations. Although the raw fluorescence intensities varied substantially across neurons, the % change in response to the same agonist did not (Table 1). The total area under the curve (arbitrary unit) was calculated from the response onset to the time point where the response returned to the baseline.

**Table 1:** Variations of Ca^2+^ responses (raw fluorescence intensities and averages, in arbitrary unit) in individual MNTB neurons induced by activation of glutamate receptors

Agonist	#Cell	Baseline (AU)	Peak (AU)	% change
NMDA	#1	34109	55716	63.3%
	#2	37961	60512	59.4%
	#3	39931	62579	56.7%
	#4	34248	57720	68.5%
	#5	29585	50901	72.1%
	#6	26341	43323	64.5%
	#7	41903	64264	53.4%
	#8	32127	50499	57.2%
	Average	34526 ± 1856 (8)	55689 ± 2504 (8)	61.3%
AMPA	#1	9910	11653	17.6%
	#2	10118	12219	20.8%
	#3	9514	11625	22.2%
	#4	10336	12755	23.4%
	#5	10629	12449	17.1%
	#6	10510	12636	20.2%
	#7	10464	12713	21.5%
	#8	9215	10715	16.3%
	#9	9844	11630	18.1%
	#10	9464	11439	20.9%
	#11	9518	11532	21.2%
	Average	9957 ± 147 (11)	11942 ± 197 (11)	19.9%
DHPG				
	#1	8665	9571	10.5%
	#2	8963	9719	8.4%
	#3	9212	10663	15.8%
	#4	9271	10268	10.8%
	#5	8333	8983	7.8%
	#6	8663	9275	7.1%
	#7	8951	9875	10.3%
	#8	8495	9512	12.0%
	#9	9343	10305	10.3%
	Average	8877 ± 120 (9)	9797 ± 179 (9)	10.4%

**Fig. 1:**
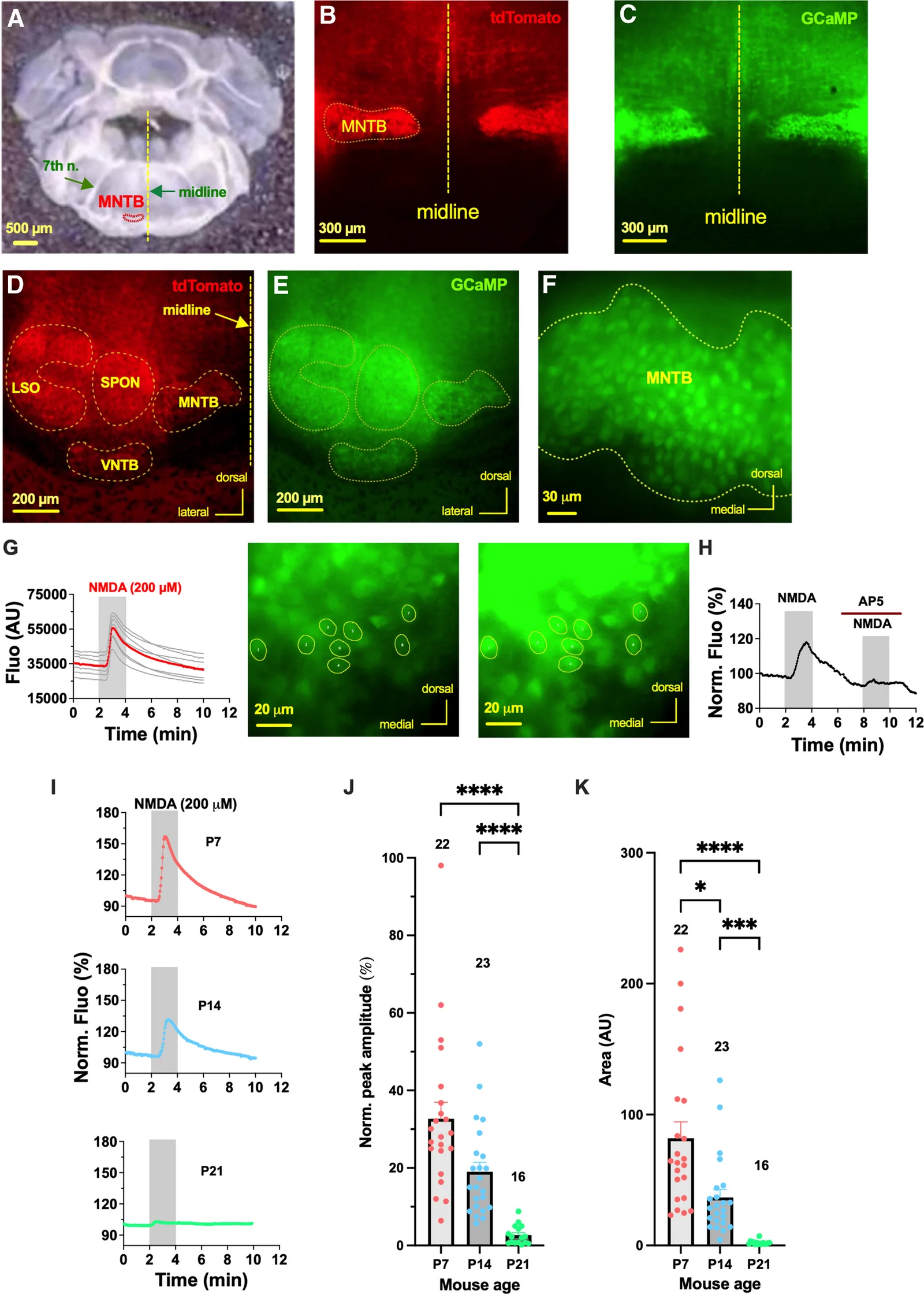
Developmental downregulation of NMDA-induced Ca^2^⁺ signaling in MNTB neurons. **A** Coronal brainstem slice showing the location of MNTB in an acutely prepared slice (P14). The MNTB can be readily identified using the 7th nerve and the midline as anatomical landmarks. **B, C** Images showing tdTomato and GCaMP fluorescence in the MNTB on both sides (P7). **D** tdTomato signal in the area containing the superior olivary complex. Strong fluorescence in the superior paraolivary nucleus (SPON) and lateral superior olive (LSO) confirms glycinergic inputs to these nuclei in the reporter mouse (P7). **E** GCaMP signal in the same area as the one in panel D. **F** Expression of the green, fluorescent Ca^2^⁺ sensor protein GCaMP6f in MNTB glycinergic neurons, visualized under 480 nm excitation in a slice obtained from a GlyT2-Cre/tdTomato-GCaMP6f transgenic mouse (P7). **G** Representative Ca^2^⁺ imaging traces from a region of interest (ROI) within the MNTB, showing Ca^2^⁺ transients of 8 individual cells (gray) and their average response (red) following bath application of NMDA (200 μM) (Gray bar). The averaged response was used to calculate the amplitude and area under the curve, representing one data point. The two images with green fluorescence show the selection of MNTB neurons at the same focus plane and the intensity of fluorescence at the baseline prior to NMDA and at the peak of the response. **H** Bath application of the competitive NMDAR antagonist AP5 (50 μM) markedly reduced the NMDA-evoked Ca^2^⁺ transients, confirming NMDAR-mediated Ca^2^⁺ signaling. **I** Representative average fluorescence traces of NMDA-induced Ca^2^⁺ transients at different postnatal ages (P7, P14, and P21), showing a progressive reduction in Ca^2^⁺ signaling with maturation. **J** Quantification of the normalized peak amplitude (%) of NMDA-evoked Ca^2^⁺ transients in MNTB neurons at different ages. The peak amplitude was highest at P7 (*N =* 22 mice), moderately reduced at P14 (*N =* 23), and nearly absent by P21 (*N =* 16), indicating a strong developmental decline. The numbers above bars represent the number of animals analyzed (N). **K** Statistical analysis of the area under the curve in arbitrary unit (AU). The area integrates the Ca^2^⁺ signaling over a time period between the start and the end of the response, providing a measure of the overall total Ca^2^⁺ influx. The total response area was large at P7, decreased significantly with age. Mean ± SEM are shown in this and subsequent figures, unless otherwise stated. * *p <* 0.05, ** *p <* 0.01, *** *p <* 0.001, **** *p <* 0.0001 (ANOVA post hoc test), and non-significant results are left blank. Norm.: normalized

**Fig. 2:**
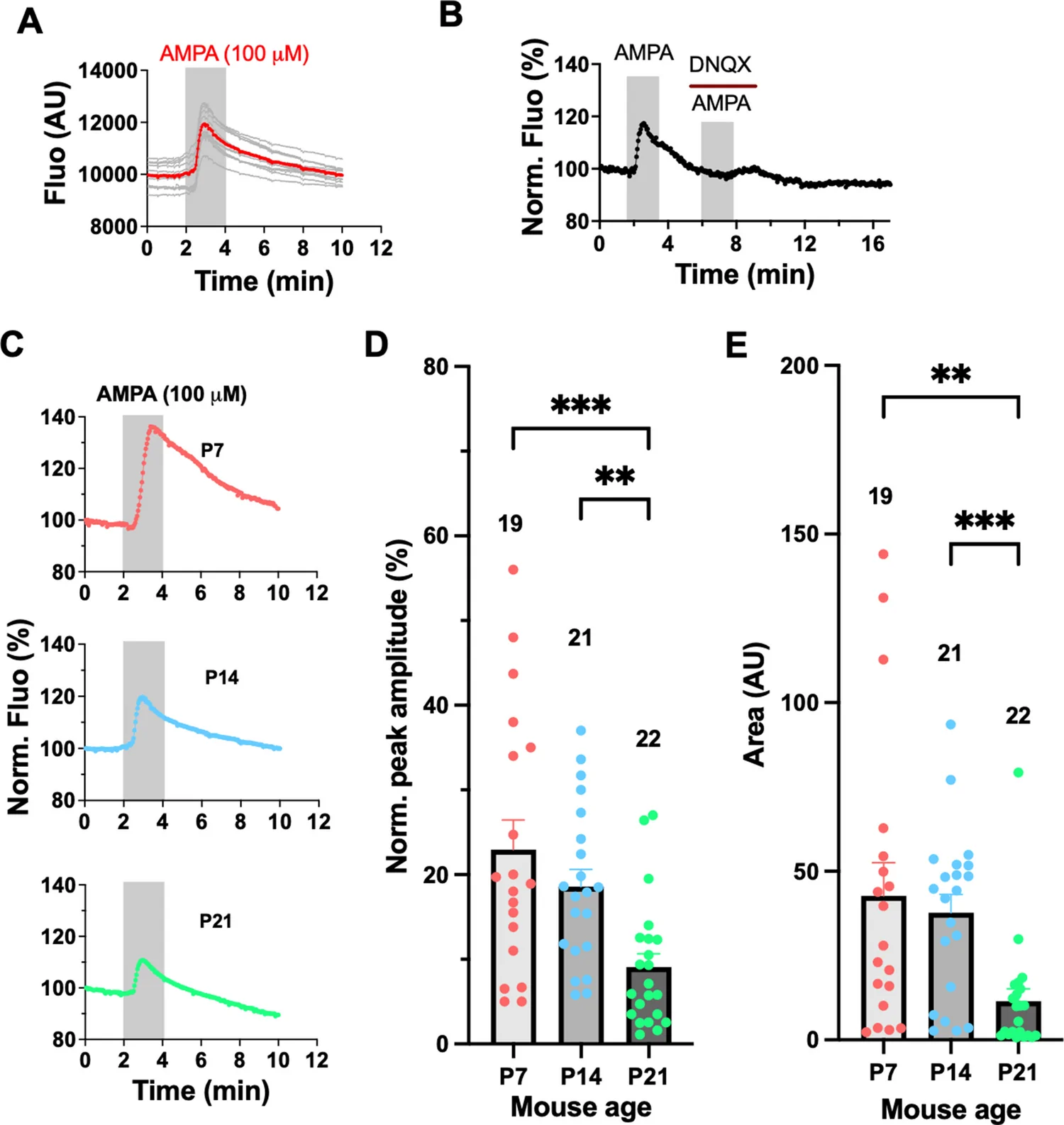
Ca^2+^ signaling mediated by activation of AMPARs decreases during postnatal development in MNTB neurons. **A** Representative Ca^2^⁺ imaging traces, showing the averaged (red) and 11 individual (gray) AMPA (100 μM)-induced Ca^2+^ responses. **B** The Ca^2+^ response to AMPA was largely abolished by the AMPA receptor antagonist DNQX (50 μM). **C** Representative normalized fluorescence traces showing AMPA-evoked Ca^2+^ responses at three developmental stages (P7, P14, and P21). Note the progressive reduction in peak amplitude and duration with increasing age. **D** Normalized peak amplitude (%) of AMPA-induced Ca^2+^ signals plotted as a function of developmental stages. The amplitude of Ca^2+^ responses reduced progressively with age, although the Ca^2+^ signaling remained relatively strong even at P21. **E** Quantification of total response area for AMPA-induced Ca.^2+^ transients at each age. Both response amplitude and area show consistently significant downregulation from P7 (*N =* 19) to P21 (*N =* 22), and from P14 (*N =* 21) to P21 (*N =* 22)

**Fig. 3:**
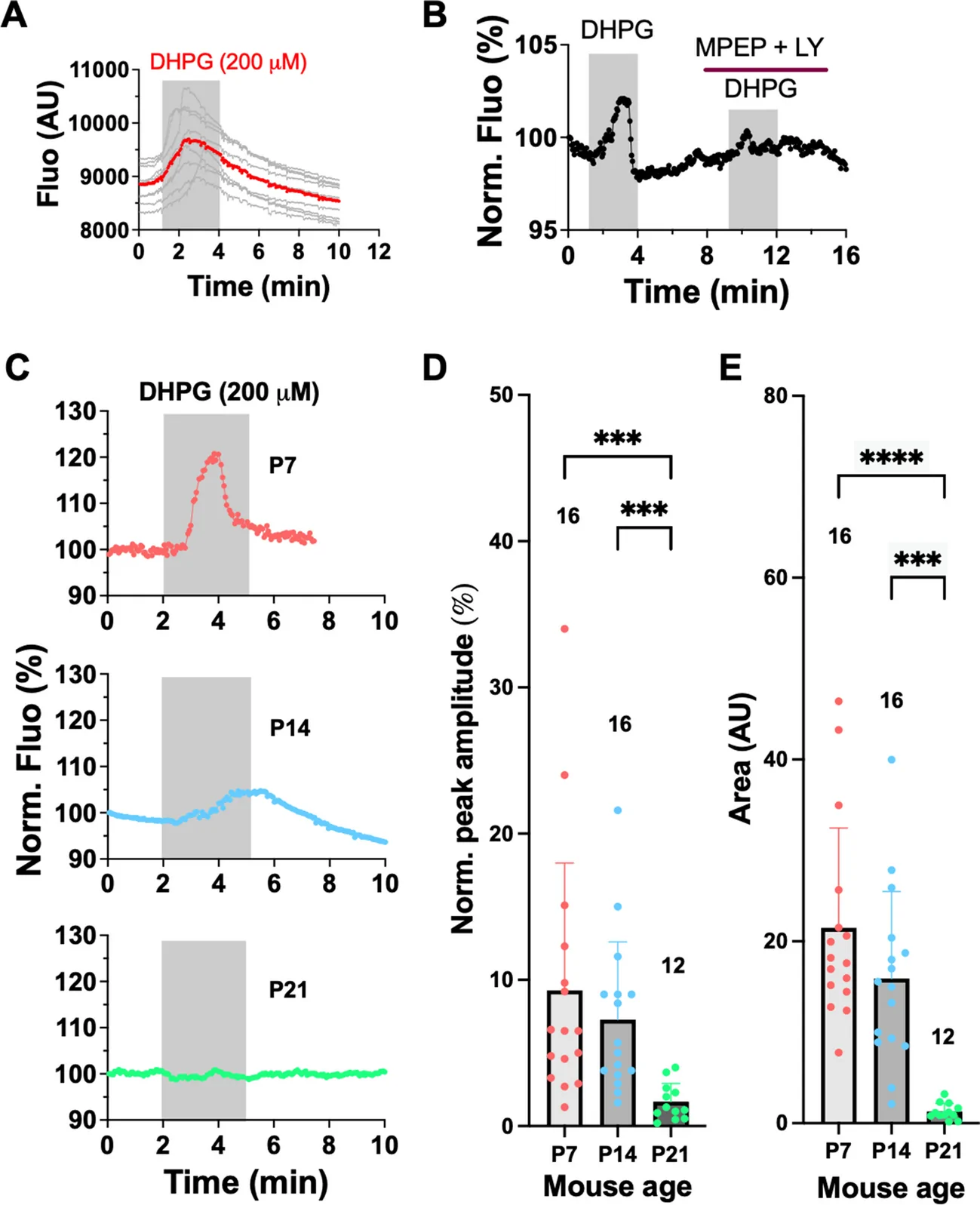
Activation of group I mGluRs by 3,5-DHPG (200 μM) evokes Ca^2+^ signaling in MNTB neurons that reduces after hearing onset and diminishes at P21. **A** Representative Ca^2^⁺ imaging traces, showing the averaged (red) and individual (gray) 3,5-DHPG (200 μM)-induced Ca^2+^ responses. **B** The Ca^2+^ response was largely abolished by the combination of LY367385, antagonist for mGluR1 (50 μM), and MPEP, antagonist for mGluR5 (1 μM). **C** Normalized fluorescence sample traces illustrating DHPG-evoked Ca^2+^ responses at P7, P14, and P21. The amplitude of the responses markedly decreased with age. **D** 3,5-DHPG-evoked peak Ca^2+^ response amplitudes showed a progressive developmental decline, with robust responses observed before hearing onset (P7, *N =* 16) that reduced thereafter (P14, *N =* 16) and were largely absent in near-mature neurons (P21, *N =* 12). **E** The overall Ca^2+^ signal area shows consistently significant developmental downregulation

For the averages, Mean ± SEM (number of cells) are reported. The numerical values represent the data in Figs. 1G, 2A, and 3A, respectively

Statistical analyses were performed, and graphs were made with GraphPad Prism10 (GraphPad Software, San Diego, CA). No obvious differences were observed between the sexes, and data from both sexes were therefore combined. Mean ± SEM (standard error of the mean) and number of animals (N) are reported, unless otherwise indicated. Data were subject to statistical analyses using ANOVA. The normality of data distribution was examined with Kolmogorov–Smirnov test. The data that passed the normality test were analyzed with parametric ANOVA, whereas data that failed the normality test were analyzed with Kruskal–Wallis test. A post hoc Dunn’s multiple comparisons test was used to compare and detect differences between two groups. Any difference with a p value less than 0.05 was considered statistically significant.

## Results

The developmental profiles of Ca^2^⁺ signaling evoked by various receptor agonists in MNTB neurons were systematically assessed using Ca^2^⁺ imaging in brain slices prepared from GlyT2-Cre/tdT-GCaMP6f mice at different postnatal ages. The MNTB was identified using the seventh cranial nerve and the midline as landmarks (Fig. 1A). We observed red fluorescence due to the expression of tdTomato in the area containing the superior olivary complex. Because we used GlyT2-Cre to drive the expression of both tdTomato and GCaMP, glycinergic cells in MNTB as well as glycinergic terminals in target nuclei such as the superior paraolivary nucleus (SPON) and lateral superior olive (LSO) display tdTomato fluorescence (Fig. 1B, D). Green fluorescent MNTB cell bodies were clearly visualized under 480-nm excitation (Fig. 1C, E, F).

### NMDA-induced Ca^2+^ Signaling is Developmentally Downregulated

NMDARs are highly permeable to Ca^2^⁺, and NMDAR-mediated Ca^2^⁺ signaling plays pivotal roles during development and synaptic plasticity [37]. In a region of interest (ROI) of the MNTB obtained from a P7 mouse, we analyzed Ca^2^⁺ signaling in 8 cells that were at about the same focus plane. Bath application of NMDA (200 µM) induced a transient increase in Ca^2^⁺ (Fig. 1G, gray traces), in a group of selected cells that are on the same focus plane. NMDA was applied in the presence of physiological concentration of Mg^2+^ (1.3 mM) in order to acquire NMDA-induced Ca^2+^ responses under the physiological conditions. To ensure rigorous data analysis, we used the average response from these cells on a single brainstem slice to represent one data point for the animal (Fig. 1G, red trace) and report the number of animals (N) rather than the number of cells (n) in population data. To confirm that the Ca^2^⁺ responses were specifically induced by activation of NMDARs, we bath-applied the competitive NMDAR antagonist AP5 (50 μM) (Fig. 1H). Under AP5 treatment, Ca^2^⁺ transients were almost completely abolished, confirming that the observed signals were indeed mediated by NMDAR activation. When NMDA was applied using the same protocol to brain slices obtained at different ages, representative traces showed robust Ca^2^⁺ responses at P7, reduced responses at P14, and minimal responses at P21 (Fig. 1I). Population data analysis revealed that the peak amplitude of these responses was highest at P7 (*N =* 22), moderate at P14 (*N =* 23), and lowest at P21 (*N =* 16) (Fig. 1J; Table 2), consistent with the well-established developmental dependence on glutamatergic NMDAR signaling in the immature auditory brainstem [22,38,39].

**Table 2:** Comparisons of Ca^2+^ responses induced by activation of glutamate receptors

Agonist Measurement	P7 (N)	P14 (N)	P21(N)	ANOVA *p*
NMDA amp (%)	32.7 ± 4.0 (22)	19.0 ± 2.0 (23)	2.7 ± 1.0 (16)	< 0.0001****
NMDA area (AU)	81.91 ± 12.47 (22)	36.68 ± 6.23 (23)	2.08 ± 0.39 (16)	< 0.0001****
AMPA amp (%)	23.0 ± 4.0 (19)	18.6 ± 2.0 (21)	9.1 ± 2.0 (22)	0.0003***
AMPA area (AU)	42.68 ± 9.89 (19)	37.72 ± 5.41 (21)	11.49 ± 3.63 (22)	0.0002***
DHPG amp (%)	9.3 ± 2.0 (16)	7.3 ± 1.0 (16)	1.7 ± 0.4 (12)	< 0.0001****
DHPG area (AU)	21.48 ± 2.75 (16)	15.90 ± 2.40 (16)	1.30 ± 0.26 (12)	< 0.0001****

Mean ± SEM (*N =* number of animals) are reported. ANOVA, *, **, ***, and ****: *p <* 0.05, 0.01, 0.001, and 0.0001, respectively

We further analyzed the total response area under the curve, which reflects both the amplitude and duration of Ca^2^⁺ elevation (Fig. 1K). At P7, NMDA produced large and sustained Ca^2^⁺ responses, resulting in a high integrated signal area. By P14, the total response area was significantly reduced compared with P7. This reduction between P7 and P14 was not significant when only the amplitude was analyzed, indicating that the decrease involved not only the magnitude but also the kinetics of Ca^2^⁺ entry through NMDARs at P14. At P21, the response area was minimal, consistent with the near absence of detectable Ca^2^⁺ transients at this stage. These findings demonstrate that NMDAR-dependent Ca^2^⁺ signaling is developmentally regulated in the MNTB, highly active during early postnatal stages and progressively downregulated as auditory experience begins and neural circuits mature. This developmental downregulation confirms a temporal restriction of NMDAR-driven Ca^2^⁺ signaling in MNTB neurons [22,24,38].

### AMPA-induced Ca^2+^ Signaling is Developmentally Downregulated but Remains Relatively Strong at Maturation

AMPARs are the primary ionotropic glutamate receptors mediating most synaptic signaling throughout development and into maturity, and AMPARs lacking the subunit GluR2 are Ca^2^⁺ permeable [40]. Such receptors are prominent in the auditory brainstem owing to their fast kinetics. Consistent with this, bath application of AMPA (100 µM) elicited robust and sustained Ca^2^⁺ transients in individual MNTB neurons (Fig. 2A). These responses were largely abolished by the selective AMPAR antagonist DNQX (50 μM) (Fig. 2B), confirming that the signals were mediated by AMPAR activation. At P7, AMPA induced strong Ca^2^⁺ responses (Fig. 2A; *N =* 19). Following the onset of hearing at P14, the magnitude of AMPA-induced Ca^2^⁺ responses showed a modest decline (*N =* 21), but the difference compared with P7 was not statistically significant. By P21, however, the AMPA-induced Ca^2^⁺ signals were significantly reduced (*N =* 22), although a residual level of Ca^2^⁺ signaling remained detectable (Fig. 2C–E; Table 2). These persistent AMPA-induced responses contrast with the near absence of NMDA-evoked Ca^2^⁺ signals at the same age, suggesting that AMPARs continue to contribute to Ca^2^⁺ influx in more mature MNTB neurons. Together, these results indicate that AMPA-induced Ca^2^⁺ signaling is strongly active during early postnatal stages but undergoes substantial downregulation after the onset of hearing, while retaining a residual component that may support synaptic function and plasticity in mature MNTB neurons.

### Gp-I mGluR Activation-induced Ca^2+^ Signaling is Developmentally Downregulated

Gp-I mGluRs include mGluR1 and mGluR5. Both members are expressed in postsynaptic MNTB neurons [32,41]. mGluR5 is also expressed in calyx of Held [32]. Activation of these GPCRs in postsynaptic cells leads to Ca^2^⁺ release from internal stores [41,42]. We first confirmed the response by Gp-I mGluR agonist 3,5-DHPG (200 µM) was induced by mGluR1/5 by using the combination of their antagonists (LY367385, 50 µM and MPEP, 1 µM) (Fig. 3A–B). We next investigated whether Ca^2^⁺ signaling induced by Gp-I mGluR activation changes across developmental stages. Bath application of the Gp-I mGluR agonist 3,5-DHPG (200 μM, for 3 min) evoked large Ca^2^⁺ transients at P7, smaller responses at P14, and diminished responses at P21 (Fig. 3C). The responses at P7 were characterized by large amplitudes, indicating strong receptor activation and subsequent Ca^2^⁺ mobilization from intracellular stores. Both the amplitude (Fig. 3D) and the response area (Fig. 3E) declined by P14 compared with P7. At P21, Ca^2^⁺ responses were nearly absent, evidenced in both peak amplitude and area values. This developmental profile closely paralleled the downregulation observed for NMDA-induced Ca^2^⁺ signaling (Fig. 1; Table 2), suggesting a coordinated reduction of multiple Ca^2^⁺ entry and release pathways during auditory circuit maturation.

### Relative Contributions to Ca^2+^ Signaling by Various Glutamate Receptors

**Fig. 4:**
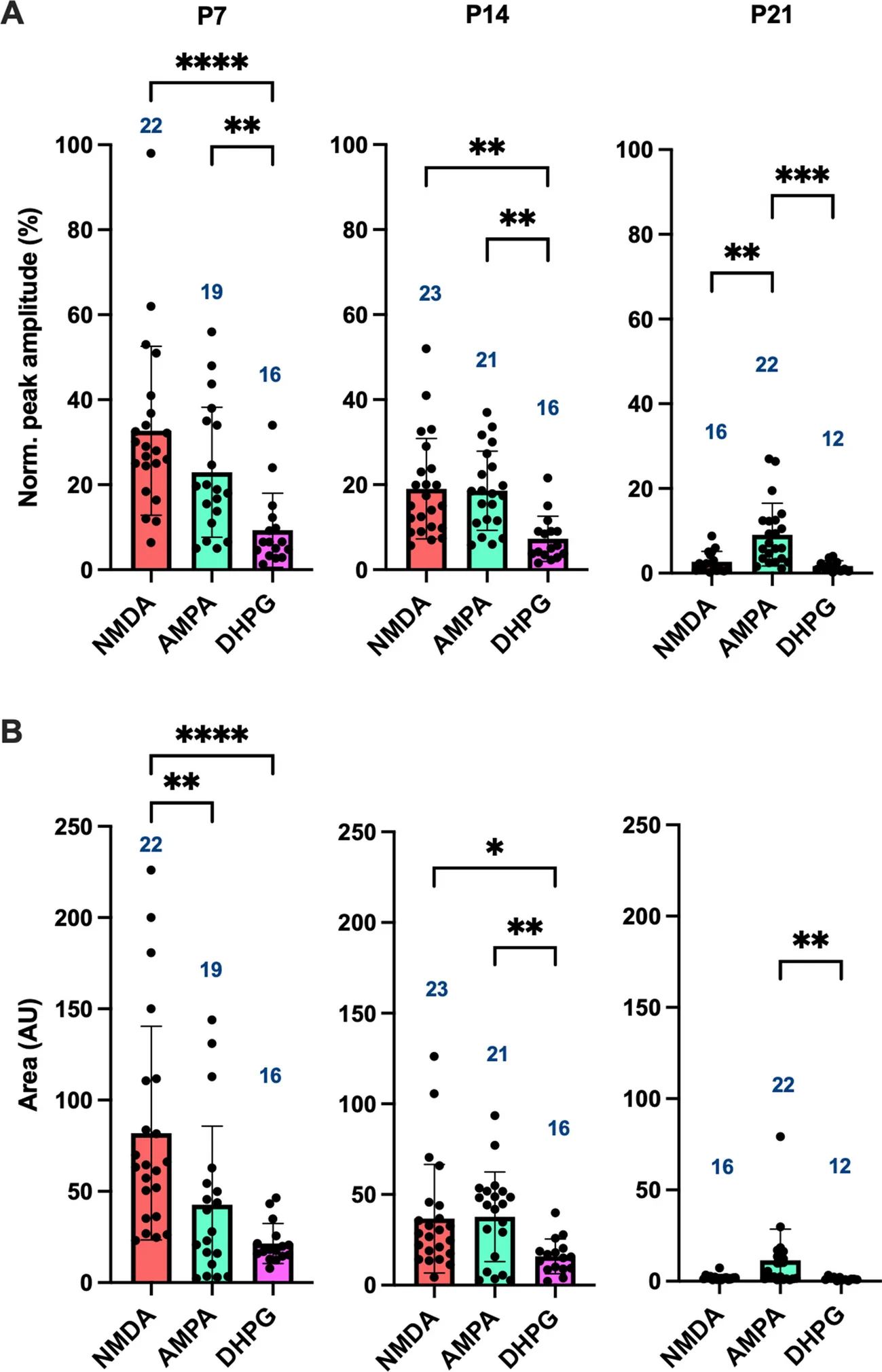
Relative contributions to Ca^2+^ signaling induced by activation of glutamate receptors in MNTB principal neurons at a particular age. **A** Quantification of normalized peak amplitude (%) evoked by saturating concentrations of NMDA, AMPA, and 3,5-DHPG at P7, P14, and P21. At P7, NMDA-evoked Ca^2+^ signals were the strongest in comparison to those evoked by AMPA or 3,5-DHPG. mGluRs contributed the least among all three age groups. At P21, AMPA-evoked Ca^2+^ peaks became the highest. **B** Consistently, total response area for each receptor type at different developmental stages shows a similar relative contributions to the Ca^2+^ signaling at a particular age. The same range for the Y-axis was used for readily comparisons

Activation of either ionotropic or metabotropic glutamate receptors by using their respective agonists at their saturating concentrations elicited Ca^2^⁺ responses in MNTB neurons, with a general trend of downregulation for each receptor activation-induced component (Figs. 1–3). To determine the relative contributions of individual receptor types, at their maximal activation levels, to the overall Ca^2^⁺ signaling, we re-analyzed the data by categorizing responses according to receptor type at each developmental stage (Fig. 4A–B). Analyses of both Ca^2^⁺ response amplitude and area revealed that at P7, the NMDA-induced response was the strongest. By P14, both NMDA- and AMPA-induced responses were reduced; however, because the downregulation of AMPA-induced responses was less pronounced, their amplitudes became comparable to those of NMDA-induced responses. By P21, this developmental trend continued, resulting in AMPA-induced responses becoming dominant and contributing the most to glutamate receptor–mediated Ca^2^⁺ signaling. In all three age groups, the Ca^2^⁺ response elicited by 3,5-DHPG was the smallest among the three receptor types.

### Synaptic Glutamatergic Activity-induced Ca^2+^ Signaling is Developmentally Downregulated

**Fig. 5:**
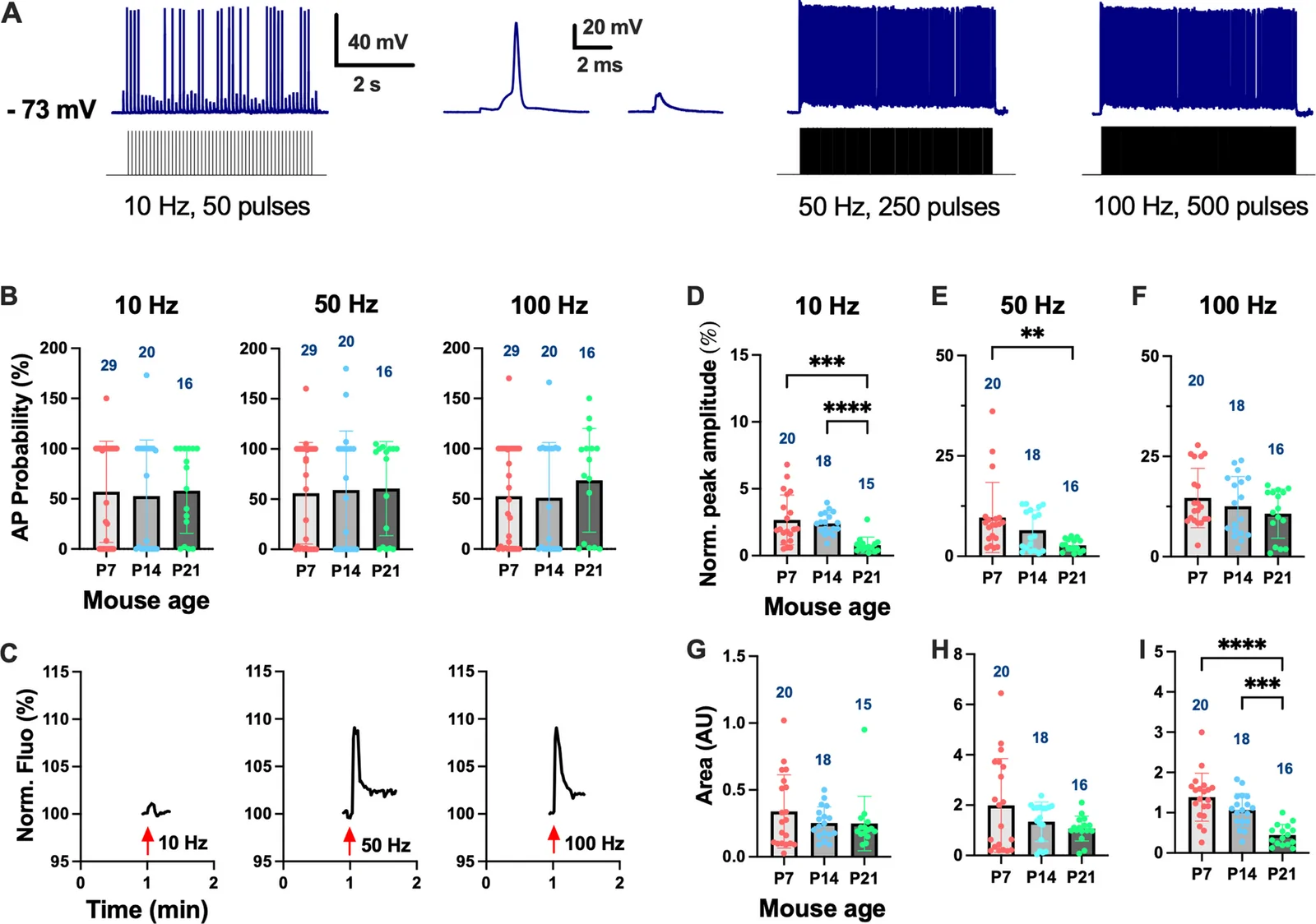
Synaptic activity–induced Ca^2+^ signaling declines during postnatal development in MNTB neurons. **A** Representative whole-cell current clamp recordings from postsynaptic MNTB principal neurons (P7) showing EPSPs and action potentials (AP) generated in response to afferent fiber stimulation at increasing frequencies (10, 50, and 100 Hz). Trains of stimuli were delivered for 5 s (50, 250, and 500 pulses, respectively). One representative AP and one EPSP are shown on the top at expanded scales. Stimulus artifacts were deleted for clarity. **B** Summary plots showing the probability of AP generation at each stimulation frequency across developmental stages (P7, *N =* 29; P14, *N =* 20; P21, *N =* 16 cells). The average AP probability remained largely unchanged across frequencies and ages, indicating stable functional synaptic connectivity and similar stimulation effectiveness in the ages tested. **C** Representative averaged Ca^2+^ traces from MNTB principal neurons at P7 in response to synaptic stimulation at various frequencies. Ca^2+^ responses increased with stimulation frequency, showing strong summation at higher frequencies. **D-F** Quantification of peak Ca^2+^ response amplitudes induced by stimulation at various frequencies. Synaptic activity induced Ca^2+^ transients were large at P7 (*N =* 20) and P14 (*N =* 18), and significantly reduced by P21 (*N =* 16), reflecting developmental downregulation of activity-dependent Ca^2+^ signaling. Note that in 1 P21 mouse, data at 10 Hz were not acquired. (**G-I**) The areas of the Ca^2+^ responses showed a consistent developmental profile with that of the amplitudes

To examine Ca^2^⁺ signaling in MNTB neurons under more physiologically relevant conditions, we performed Ca^2^⁺ imaging during electrical activation of the glutamatergic input from the contralateral cochlear nucleus. To assess the effectiveness of synaptic stimulation, we recorded postsynaptic responses using whole-cell current-clamp recordings from MNTB neurons while delivering stimulus trains at three frequencies (10, 50, and 100 Hz), each lasting 5 s (50, 250, and 500 pulses, respectively). The stimulus intensity (0.5–0.7 mA) was chosen based on parameters routinely used in our physiological experiments to activate the synaptic input [32,43]. Under such stimulation conditions, EPSPs and/or APs were evoked (Fig. 5A). About half of the cells exhibited high AP probability, while the others exhibited high failure rate (no AP generation but with EPSPs present), owing to the nature of the synaptic stimulation paradigm. In some cells, a single stimulus pulse within the train stimulation protocol (10, 50, or 100 Hz) elicited more than one AP, resulting in firing probabilities exceeding 100%. On average at the cell population level, there were no significant differences in AP probability among the three age groups, with the mean at about 50% (Fig. 5B; P7, *N =* 29; P14, *N =* 20; P21, *N =* 16), indicating that the stimulation protocol was similarly effective in activating the glutamatergic pathway across these 3 ages. The bimodal distribution of firing probabilities likely reflects differences in the success of stimulating the calyceal input. Depending on the stimulation site and the recorded neuron, one subset of cells responded with APs that followed the stimulation with high fidelity. In contrast, another subset exhibited only EPSPs without generating APs, possibly because stimulation activated non-calyceal rather than calyceal inputs. Because non-calyceal inputs are relatively weak [44,45], they may be insufficient to drive MNTB neurons to fire APs.

Next, we applied the same synaptic stimulation protocols and monitored Ca^2^⁺ signaling in MNTB neurons. This approach was intended to evoke Ca^2^⁺ increases in response to receptor activation by endogenous neurotransmitters, presumably glutamate. We observed stimulus frequency–dependent Ca^2^⁺ increases in MNTB neurons, with response amplitudes increasing as the stimulation frequency increased (Fig. 5C). In most cases, the Ca^2^⁺ responses appeared to reach saturation at 50 Hz stimulation. When normalized to the peak amplitude of electrically evoked Ca^2^⁺ responses, the response amplitude showed age-dependent downregulation (Fig. 5D–F and Table 3; P7, *N =* 20; P14, *N =* 18; P21, *N =* 15–16). Analysis of the response area revealed similar pattern of developmental downregulation of the Ca^2^⁺ responses (Fig. 5G–I and Table 3). Regardless of the stimulus frequency, consistent with the results of bath application of glutamatergic receptor agonists, the Ca^2^⁺ responses were the strongest at P7, reduced at P14 and the weakest at P21.

**Table 3:** Comparisons of Ca^2+^ responses evoked by synaptic stimulation at different frequencies

Freq (Hz)	Measurement	P7 (N)	P14 (N)	P21 (N)	ANOVA *p*
10	amp (%)	2.7 ± 0.4 (20)	2.4 ± 0.2 (18)	8.0 ± 0.2 (15)	< 0.0001****
10	area (AU)	0.34 ± 0.06 (20)	0.25 ± 0.03 (18)	0.25 ± 0.05 (15)	0.7346
50	amp (%)	9.7 ± 2.0 (20)	6.5 ± 1.0 (18)	2.7 ± 0.4 (16)	0.0023**
50	area (AU)	1.99 ± 0.42 (20)	1.34 ± 0.18 (18)	1.07 ± 0.12 (16)	0.3324
100	amp (%)	14.6 ± 2.0 (20)	12.6 ± 2.0 (18)	10.7 ± 2.0 (16)	0.3653
100	area (AU)	1.38 ± 0.13 (20)	1.07 ± 0.10 (18)	0.44 ± 0.07 (16)	< 0.0001****

Mean ± SEM (*N =* number of animals) are reported. ANOVA, *, **, ***, and ****: *p <* 0.05, 0.01, 0.001, and 0.0001, respectively

### GABA/Glycine-induced Ca^2+^ Signaling is Presented in Neonatal Mice and Diminishes Nearly Completely by P7

**Fig. 6:**
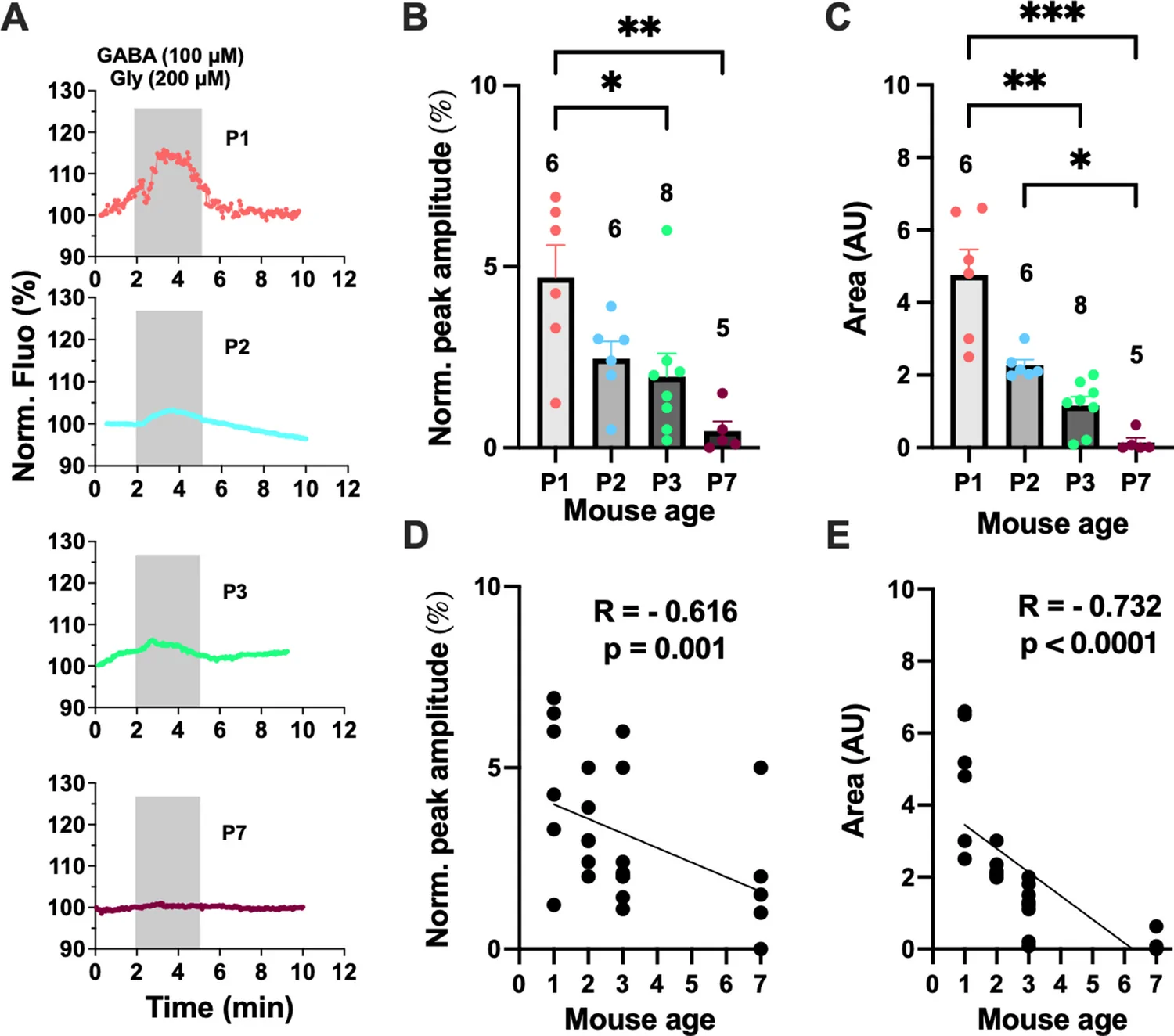
GABA/glycine–induced Ca^2+^ signaling is restricted to the neonatal period in MNTB principal neurons. **A** Normalized sample traces showing Ca^2+^ responses evoked by co-application of GABA (100 μM) and glycine (200 μM) at P1, P2, P3, and P7. A small Ca^2+^ response was observed at P1, diminished at P2/3, and was absent at P7. **B, C** The peak Ca.^2+^ response and the total response area evoked by GABA/glycine co-application showed a trend of downregulation across developmental stages (P1–P3). **D**, **E** Linear regression analyses showing a significant negative relationship (downregulation) between peak amplitude and age (R = −0.616, p = 0.001), and between response area and age (R = −0.732, *p <* 0.0001)

In early development, due to a relatively high intracellular Cl⁻ concentration, activation of GABA_A_ receptors (GABA_A_R) and glycine receptors (GlyR) causes membrane depolarization and even spike generation [46], which in turn can activate VGCCs and increase intracellular Ca^2^⁺ levels. To investigate this mechanism in MNTB neurons, we examined Ca^2^⁺ signaling evoked by GABA_A_R and GlyR activation in neonatal mice. Concurrent bath application of GABA (100 μM) and glycine (200 μM) elicited a transient increase in intracellular Ca^2^⁺ in MNTB neurons within a very narrow developmental window (Fig. 6A). Ca^2^⁺ transients were observed at P1, became smaller and less frequent at P2, and further reduced by P3. By P7, no responses were detectable. Because the overall responses were small, we did not attempt to separate the contributions of GABA_A_Rs versus GlyRs, and assume that GABA_A_Rs are the primary mediators because they are more dominant than GlyRs in early developmental stage of MNTB [47,48]. One-way ANOVA revealed statistically significant differences in the response amplitude or area between P1 and P3/P7 (Fig. 6B, C; P1, *N =* 6; P2, *N =* 6; P3, *N =* 8; P7, *N =* 5). Consistently, linear regression analysis of response amplitude and area versus mouse age showed a significant negative correlation between the Ca^2+^ response and mouse age (Fig. 6D, E), indicating a rapid downregulation of Ca^2^⁺ transients mediated by GABA_A_R and GlyR activation during early postnatal development.

## Discussion

**Fig. 7:**
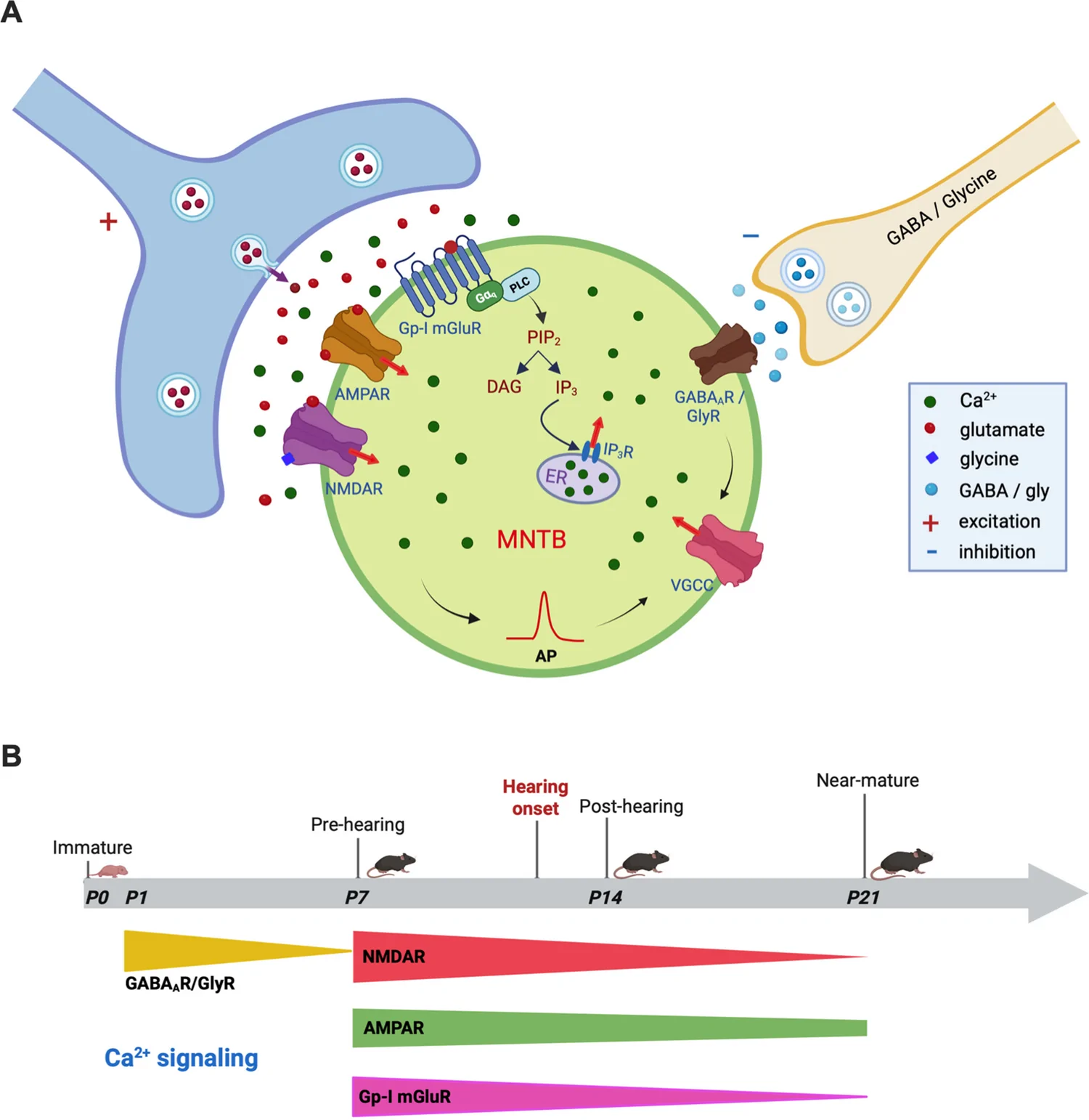
Summary of developmental changes in Ca^2+^ signaling in MNTB principal neurons. **A** Schematic illustration showing the major receptor systems contributing to postsynaptic Ca^2+^ signaling in MNTB neurons. Ca^2+^ influx is largely mediated by activation of ionotropic glutamate receptors (NMDARs, AMPARs), Gp-I mGluRs, and VGCCs. Combined activation of GABA_A_Rs and GlyRs evokes Ca^2+^ responses only during neonatal stages. **B** Developmental profile summarizing the relative contributions of each receptor system to Ca^2+^ signaling from birth (P1) to near maturation (P21). The illustration was made in BioRender

Using GCaMP-based Ca^2^⁺ imaging and electrophysiology, we systematically characterized receptor-specific contributions to intracellular Ca^2^⁺ dynamics during critical periods of auditory circuit development, with a particular focus on Ca^2^⁺ signaling induced by activation of glutamate receptors in mouse MNTB neurons. We also identified a previously unrecognized phenomenon in which inhibitory receptor activation elicited Ca^2^⁺ responses in neonatal MNTB neurons; these responses nearly completely disappeared during the first postnatal week. Finally, by applying electrical stimulation to the major excitatory afferent pathway, we examined synaptic activity–induced Ca^2^⁺ dynamics in MNTB neurons. Overall, before hearing onset, Ca^2^⁺ signaling was robust and initiated by multiple glutamatergic receptors. After hearing onset, Ca^2^⁺ signaling, particularly the NMDA- and 3,5-DHPG-induced components, was markedly reduced (Fig. 7A–B), suggesting a crucial role for Ca^2^⁺ signaling in auditory brainstem circuit formation.

### Development of NMDA-induced Ca^2+^ Signaling

Several mechanisms likely contribute to the high level of NMDA-induced Ca^2^⁺ signaling before hearing onset and its subsequent developmental downregulation. First, NMDARs are abundantly expressed early in development, particularly in regions undergoing synaptogenesis and refinement. At the calyx of Held–MNTB synapse, NMDARs exhibit substantial developmental changes, with high expression from P3–P4 until hearing onset (P10–12), followed by marked downregulation in mature neurons [22,49]. Evoked NMDAR-EPSCs increase until P11–12, then decline sharply by P16 [21,50]. Second, NMDARs are heteromeric channels composed of two GluN1 and two GluN2/3 subunits [51]. Early postnatal NMDARs are enriched in GluN2B, which confers slower kinetics and greater Ca^2^⁺ permeability, thereby prolonging Ca^2^⁺ influx. During later development, GluN2B is downregulated and replaced by GluN2C, supporting faster, low-plasticity synaptic transmission [22,24]. Third, the voltage-dependent Mg^2^⁺ block of NMDARs is more effective in mature neurons and is therefore less readily relieved by depolarization-dependent unblocking [52], which further augments the downregulation of NMDAR-mediated Ca^2^⁺ signaling.

### Development of AMPA-induced Ca^2+^ Signaling

AMPA-induced Ca^2^⁺ signaling was also developmentally downregulated, though to a much lesser extent than NMDA-induced responses. AMPA-induced Ca^2^⁺ responses remained relatively strong at P21. This pattern is consistent with the dependence of AMPAR-mediated Ca^2^⁺ responses on subunit composition. AMPARs are heterotetramers composed of GluA1–4 subunits, and only receptors lacking GluA2 are Ca^2^⁺-permeable (CP-AMPARs) [53]. CP-AMPARs are prevalent early in development, when GluA2 expression is low or unedited (around P4–P5), enabling high Ca^2^⁺ influx when activated [19]. By maturation (P30–P34), Ca^2^⁺ entry through CP-AMPARs is greatly reduced [19] likely due to subunit switch [54]. As the auditory brainstem matures for sound-driven activity, GluA2 expression increases, reducing Ca^2^⁺ permeability of AMPARs [38,50,55]. This transition in subunit components promotes synaptic stability and precise temporal coding [56]. Although AMPAR-EPSCs increase in amplitude during development, they establish sharper kinetics to suit for fast temporal processing [22], which may limit the time window for Ca^2^⁺ influx via the already reduced CP-AMPARs. Another factor that may contribute to the less extensive downregulation of AMPA-induced Ca^2^⁺ signaling after hearing onset, compared with NMDA-induced Ca^2^⁺ signaling, is differential AMPAR desensitization. Prior to hearing onset (P8–10), AMPAR desensitization in MNTB neurons is prominent, whereas at P16–18, desensitization is absent [57]. Therefore, when AMPA was bath-applied to the MNTB in P7/8 mice, rapid desensitization likely reduced Ca^2^⁺ influx through these receptors. In contrast, in older age groups, the absence of desensitization would allow each AMPAR to permit relatively greater Ca^2^⁺ influx. This effect may partially counteract other factors contributing to the downregulation of AMPAR-mediated Ca^2^⁺ signaling, although the overall trend still reflects reduced signaling with maturation.

### Development of 3,5-DHPG-induced Ca^2+^ Signaling

Gp-I mGluRs are widely expressed in brainstem nuclei, where they regulate neuronal excitability [58–60]. In the MNTB, Gp-I mGluRs are located on postsynaptic neurons [32,41]. These receptors couple to G_q_/G_11_ proteins, leading to activation of inositol trisphosphate (IP_3_) and release of Ca^2^⁺ from intracellular stores [61]. In immature MNTB neurons, such signaling may regulate synaptogenesis, excitability, and refinement of the calyx of Held synapse [62]. In addition, presynaptic mGluR5 is expressed at the calyx of Held, and its activation enhances spontaneous glutamate release and in some cells causes spike generation [32,63]. This enhanced release may lead to increases in intracellular Ca^2^⁺ via activation of AMPARs, NMDARs, and VGCCs. Because 3,5-DHPG-induced Ca^2+^ responses are small in absolute amplitude, further experiments using pharmacological blockers to tease out the contributions of these potential mechanisms were not pursued. In this study, we show that 3,5-DHPG triggered strong Ca^2^⁺ signals in MNTB neurons at early postnatal stages, followed by a marked reduction after hearing onset. Similar observations have been reported in other auditory structures, including the rat central nucleus of the inferior colliculus (highest at P6, reduced by P13–P17) [64] and neonatal mouse lateral superior olivary (LSO) neurons (P0–P5), where responses declined and were mostly absent by P17–P19 [58].

The mechanisms underlying the developmental reduction in 3,5-DHPG-induced Ca^2^⁺ signaling in MNTB neurons remain unclear. One possibility is downregulation of mGluR1/5 expression. Postsynaptic mGluR1 has been detected in rat MNTB at P11–P14 [41], and mGluR5 in mouse MNTB at P14–P21 [32]. However, the contribution of this mechanism may be limited, because electrophysiological data show no difference in the inhibition of MNTB eEPSCs by 3,5-DHPG between two age groups comparable to those examined here (P5–P7 vs. P12–P14) [41]. Therefore, the overall extent of combined mGluR1/5 downregulation may be small. This could occur even if one receptor subtype is downregulated while the other is compensatorily upregulated. Additional mechanisms could include reduced expression or sensitivity of IP_3_ receptors, or decreased coupling efficiency of mGluRs to intracellular signaling pathways. Future work assessing protein-level expression of mGluR1, mGluR5, and IP_3_Rs in MNTB neurons will help clarify the contributions of these mechanisms. Other mGluRs such as groups II and III members are unlikely to cause Ca^2+^ signaling in postsynaptic MNTB neurons, because functional presynaptic but not postsynaptic expression of these receptors is reported [65,66].

### Development of Synaptic Glutamatergic Activity-induced Ca^2+^ Signaling

The calyceal input in rodents starts formation early at ~ P3 and establish 1:1 innervation of the calyx-MNTB synapse in most neurons by P9, as revealed by volume electron microscopy [67,68]. On average, our electrical stimulation applied to the trapezoid body at the midline of a slice triggered spike firing without significant differences in probability across the frequencies tested (up to 100 Hz) in the 3 age groups. Despite the similarity in the firing probability, the electrical stimulation reliably evoked robust Ca^2^⁺ transients in MNTB neurons during early postnatal stages which progressively declined with age. This reduction was consistent across all stimulus frequencies tested (10, 50, and 100 Hz). Synaptically evoked Ca^2^⁺ signals are presumbally mediated by a combination of NMDARs, AMPARs, Gp-I mGluRs, and VGCCs, and their developmental trajectory reflects changes in synaptic properties, receptor composition, and intracellular Ca^2^⁺ handling during brainstem maturation. Ca^2^⁺ responses caused by activation of these receptors were consistently downregulated during development, making the overall trend of decline in glutamatergic pathway–mediated Ca^2^⁺ signaling unsurprising. Although intrinsic neuronal properties may affect Ca^2+^ responses via VGCCs [25], most of the key intrinsic electrical properties such as input resistance and threshold current experience dramatic changes only during the first week after birth and become relatively stable afterwards [48,69]. Therefore, contributions of developing intrinsic electrical properties to the differential Ca^2+^ signaling studied in the animals we used are expected to be minimal. Finally, changes in the expression levels of Ca^2^⁺-buffering proteins (calretinin, parvalbumin, and calbindin) may contribute to the downregulation of Ca^2^⁺ signaling in MNTB neurons. These proteins bind free intracellular Ca^2^⁺ and rapidly reduce its concentration, thereby preventing Ca^2^⁺ overload and potential excitotoxicity [70]. During development, an upregulation of Ca^2^⁺-buffering proteins is expected as neurons mature and exhibit increased firing activity. In MNTB neurons, parvalbumin is developmentally upregulated from P6 to P14 [71]. Calbindin is first detected around P8 and increases further in more mature MNTB neurons (P18–P28) [72]. Together, these developmental changes may narrow the temporal window of free Ca^2^⁺ elevation in MNTB neurons of older animals, thereby contributing to the downregulation of Ca^2^⁺ signaling.

### Transient Ca^2+^ Signaling Induced by Activation of Glycine/GABA Receptors

GABA_A_Rs and GlyRs are typically localized on postsynaptic membranes and mediate inhibition in mature neurons including MNTB [73]. However, during early development their activation produces depolarization because of the high intracellular Cl⁻ concentration [46]. A rapid shift of the depolarizing GABA/glycine responses toward hyperpolarization occurs in the first postnatal week. Our results demonstrate that in neonatal mice, activation of GlyRs and GABA_A_Rs elicited transient Ca^2^⁺ responses in immature MNTB neurons at birth (P1), but these responses diminished rapidly by P3. At P7, such responses nearly completely disappeared. This pattern is consistent with previous studies in the LSO, where GlyR/GABA_A_R activation increased intracellular Ca^2^⁺ during the first postnatal week, with responses declining in the second postnatal week [74–76]. By the second week, the MNTB–LSO synapse switches to hyperpolarizing transmission, and GlyR/GABA_A_R activation decreases intracellular Ca^2^⁺ [75,77,78]. The mechanisms underlying this early excitatory Ca^2^⁺ signaling likely involve VGCC activation. Depolarization and potential spike generation induced by GlyR/GABA_A_R activation opens VGCCs, permitting extracellular Ca^2^⁺ influx and triggering downstream cellular processes [77,79,80].

We used a combined application of GABA and glycine in this set of experiments. Notably, during early development synaptic inputs to the MNTB induced by GABA and glycine undergo not only a transmitter-type switch from primarily GABA to glycine [47,48] but also a depolarization-to-hyperpolarization switch that occurs at ages different from those in the LSO [81]. In P5–P7 rat MNTB, GABA_A_Rs are the primary Cl⁻-permeable receptors; after P8 the faster GlyRs become more dominant [47]. The depolarization-to-hyperpolarization transition in rat MNTB may not occur until around P10 (Löhrke et al., 2005). Gramicidin perforated-patch recordings indicate depolarizing responses in P5–P7 MNTB that are not suprathreshold, and hyperpolarizing responses by P14–P15 [47]. In P7–P8 mice, although GABA/glycine may still depolarize MNTB neurons, the depolarization could be too small to activate VGCCs and therefore may not produce a detectable Ca^2^⁺ influx.

### Caveats of our Approaches

We recognize three caveats in our approaches. First, bath application of transmitter receptor agonists does not reflect faithfully the physiological synaptic activation of receptors by glutamate in vivo. Although we observed robust Ca^2^⁺ signaling, it remains unknown how strong the in vivo Ca^2^⁺ responses would be. Therefore, our conclusions regarding the developmental regulation of Ca^2^⁺ signaling are based on the assumption of either full activation or an equivalent proportion of receptor activation. Second, in our experiments, VGCCs were not blocked. Electrophysiological recordings revealed occasional spike generation upon application of each of the agonists, with varying spike probabilities at different ages (data not shown; NMDA: 4/5, 3/6, and 0/8 cells at P7, P14, and P21, respectively; AMPA: 1/4, 0/4, and 0/5 cells at P7, P14, and P21, respectively; for 3,5-DHPG, 2/10 cells in 2–3 weeks old mice [32]). Therefore, the Ca^2^⁺ signaling evoked by glutamate receptor agonists may include contributions by VGCCs. This contribution is likely to be greatest at the youngest ages examined, because MNTB neurons exhibit a high input resistance at this early developmental stage [69]. High input resistance is generally associated with increased neuronal excitability or lower threshold current [48], making neurons more likely to generate APs. Furthermore, VGCCs comprise multiple subtypes whose expression and functional contributions change during postnatal development. Consequently, the extent to which VGCCs contribute to the measured Ca^2^⁺ signals is also likely to vary with age. Leao et al. [82] showed that multiple types of VGCCs (L-, N-, and P/Q-type) are present in MNTB neurons from P12–P14 mice and contribute roughly similar proportions of the total VGCC current. Although L-type channels are expressed at the lowest level among these VGCC subtypes, they may nevertheless contribute disproportionately to Ca^2^⁺ influx because of their relatively non-inactivating properties. Such L-type VGCC-mediated Ca^2^⁺ signaling, acting through distinct molecular pathways [83,84], may play important roles in the regulation of gene expression during development, analogous to the well-established role of NMDAR-mediated signaling [85]. Finally, our comparisons across developmental ages could not explicitly account for the tonotopic organization of the MNTB. MNTB neurons are arranged in a mediolateral tonotopic gradient, with medial regions responding preferentially to high-frequency sounds and lateral regions to low-frequency sounds [16,86]. This organization is established before hearing onset [38,87,88] and are associated with maturation of VGCCs [89]. In mice at P7 or younger, the imaging ROIs encompassed almost the entire MNTB, such that Ca^2^⁺ responses were sampled across almost the full tonotopic axis and any tonotopic effects would be expected to average out; in contrast, in older mice, the ROIs covered most of the lateral but not the medial portions of the MNTB due to limitations of the imaging field, leading to underrepresentation of high-frequency regions in these age groups.

### Functional Implications

Functionally, the developmental trajectories of Ca^2+^ signaling in MNTB neurons mediated by glutamate receptors are presumably tied to the development of physiological properties to suit their cellular functions in temporal processing and sound localization. The developmental downregulation of NMDA-induced Ca^2^⁺ signaling supports the transition from a plastic, developing circuit to a mature network optimized for high-speed, temporally precise transmission required for sound localization. Hearing experience facilitates this transition because cochlear ablation before hearing onset delays the downregulation of NMDAR-EPSCs [21,22]. Suppression of prolonged NMDAR-mediated Ca^2^⁺ influx may also protect against excitotoxicity and excessive synaptic modulation, thereby preserving the fidelity of MNTB output. As AMPARs become the predominant mediators of excitatory transmission in mature MNTB synapses, reliance on NMDARs for postsynaptic excitation and Ca^2^⁺ influx diminishes. Similarly, during early development, Ca^2^⁺ influx through AMPARs plays important roles in synaptogenesis and neuronal circuit formation [90]. The transient presence of CP-AMPARs during early development allows MNTB neurons to respond to synaptic activity with Ca^2^⁺-dependent plasticity and undergo structural and functional maturation. With development, CP-AMPARs are replaced by GluA2-containing, CP-AMPARs, reflecting the shift toward high-speed, reliable neurotransmission necessary for precise auditory processing and sound localization. Downregulation of CP-AMPAR-mediated Ca^2^⁺ signaling may further protect neurons from Ca^2^⁺-induced excitotoxicity [19,40], particularly during high-frequency activity in auditory circuits. Gp-I mGluRs may contribute to neural circuit formation by enhancing spontaneous glutamate release onto MNTB neurons [32] and generating temporally patterned spike activity [63]. Such activity may reinforce spontaneous cochlea-driven firing prior to hearing onset [91] and play critical roles in neurodevelopment via the Ca^2^⁺ signaling induced by mGluRs.

## Data Availability

The datasets generated for this study are available on request to the corresponding author.
